# Structure-guided optimization of small molecules targeting the yeast casein kinase, Yck2, as a therapeutic strategy to combat Candida albicans

**DOI:** 10.21203/rs.3.rs-5524306/v1

**Published:** 2025-01-08

**Authors:** Leah Cowen, Emily Puumala, Meganathan Nandakumar, Bonnie Yiu, Peter Stogios, Benjamin Strickland, Robert Zarnowski, Xiaoyu Wang, Noelle Williams, Alexei Savchenko, David Andes, Nicole Robbins, Luke Whitesell, Timothy Willson

**Affiliations:** University of Toronto; University of Toronto; University of North Carolina at Chapel Hill; University of Toronto; University of Toronoto; University of North Carolina at Chapel Hill; University of Wisconsin-Madison; UT Southwestern Medical Center; The University of Texas Southwestern Medical Center at Dallas; University of Calgary; University of Wisconsin; University of Toronto; University of Toronto; University of North Carolina at Chapel Hill

## Abstract

*Candida albicans* is the most common cause of life-threatening fungal infection in the developed world but remains a therapeutic challenge. Protein kinases have been rewarding drug targets across diverse indications but remain untapped for antifungal development. Previously, screening kinase inhibitors against *C. albicans* revealed a 2,3-aryl-pyrazolopyridine, GW461484A (GW), which targets casein kinase 1 (CK1) family member Yck2. Here, we report optimization of GW via two complementary approaches, synthesis of bioisosteres possessing an imidazo[1,2-a]pyridine core, and R-group substitution of GW’s pyrazolo[1,5-a]pyridine core. Characterization of compounds synthesized revealed two 6-cyano derivatives with improved pharmacological properties that retained whole-cell bioactivity and selectivity for fungal Yck2 compared to human CK1α. Efficacy studies in mice indicated both analogs possess single-agent activity against *C. albicans* resistant to first-line echinocandin antifungals and potentiate non-curative echinocandin treatment. Results validate Yck2 as an antifungal target and encourage further development of inhibitors acting by this previously unexploited mode of action.

## Introduction

Fungi kill an estimated 2.5 million individuals annually, with *Candida* species being a leading cause of invasive disease. *Candida albicans* currently reigns as the most common cause of *Candida* infection with mortality rates often exceeding 40% despite therapeutic intervention.^1^ Treatment of systemic fungal infections is becoming increasingly difficult because only four front-line antifungal classes are available to treat invasive mycoses (azoles, polyenes, the pyrimidine 5-fluorocytosine, and echinocandins) with each of these classes suffering from one or more problems due to host toxicity, narrow spectrum of activity, and/or resistance development.^2–6^ Furthermore, the number of individuals susceptible to fungal infections has been steadily increasing since the 1950s, as the use of cancer chemotherapeutics and other immunosuppressive agents has become more common, and the HIV-1 pandemic has left large populations severely immunocompromised.^1^ To date, over 150,000 transplantation procedures occur per year, further increasing the global immunosuppressed population, while the widespread use of antifungal prophylaxis, in turn drives the evolution of resistance.^7,8^ These factors necessitate a coordinated response by researchers, clinicians, public health agencies, and the pharmaceutical industry as the burden of fungal disease continues to grow. This aligns with recent calls to action, including the World Health Organization’s (WHOs) Fungal Priority Pathogens list, which aims to drive research and policy interventions to address antifungal resistance and fungal disease.^9–11^

Current efforts to expand the clinical armamentarium of antifungals include development of new members of existing drug classes, identification of novel molecules targeting previously unexploited target pathways, and the exploration of antifungal immunotherapies. Fungal protein kinases are important for growth, cell signalling, proliferation, and the response to environmental stressors, making them attractive drug targets despite their broad conservation across the eukaryotic kingdom. While no kinase inhibitors are currently approved to treat fungal infection, their potential use as either single agents or potentiators of existing antifungals has been proposed.^12–15^ Foundational work from our research group supports this approach, as a screen of public-domain human kinase inhibitors revealed a synthetic small molecule, GW461484A (GW), with single-agent and echinocandin-potentiating activity against *C. albicans* and the evolutionary related and emerging fungal pathogen *Candida auris*.^12^ Using chemical-genomic approaches, we established the primary target of this 2,3-aryl-pyrazolopyridine compound is the *C. albicans* casein kinase Yck2, a member of the eukaryotic casein kinase 1 (CK1) family.^12,16^ Yck2 is required for growth of *C. albicans* under host-relevant conditions, plays a key role in governing echinocandin resistance, and enables *C. albicans* virulence in both immunocompetent and immunocompromised mice.^12,16^ GW binds to the kinase’s ATP binding pocket by interacting with multiple hydrophobic residues, while also engaging with the flexible glycine-rich loop (P-loop)^12^. The Yck2 P-loop is not well conserved with human CK1 orthologues,^12^ potentially allowing for fungal target selectivity. However, *in vivo* testing of GW has been precluded because extensive cytochrome P450 (CYP450)-mediated oxidative metabolism by liver microsomes makes it unsuitable for systemic administration.

Here, we report a multidisciplinary, structure-guided effort to generate analogs of GW with maintained or improved antifungal potency and selectivity, while reducing susceptibility to hepatic P450 metabolism. Guided by the co-structure of GW with Yck2 and its predicted hotspots for P450 metabolism, two series of molecules were synthesized. One set involved structure-guided substitutions on the parent pyrazolo[1,5-a]pyridine scaffold while the second was based on bioisosteres containing an imidazo[1,2-a]pyridine core heterocycle. Amongst the suite of compounds synthesized, two 6-cyano-substituted compounds were found to possess markedly improved whole animal pharmacology and demonstrated promising antifungal activity in a neutropenic mouse model of systemic infection by echinocandin-resistant *C. albicans*.

## Results

### Generation of two sets of molecules to improve pharmacological properties of GW

**Figure 1:**
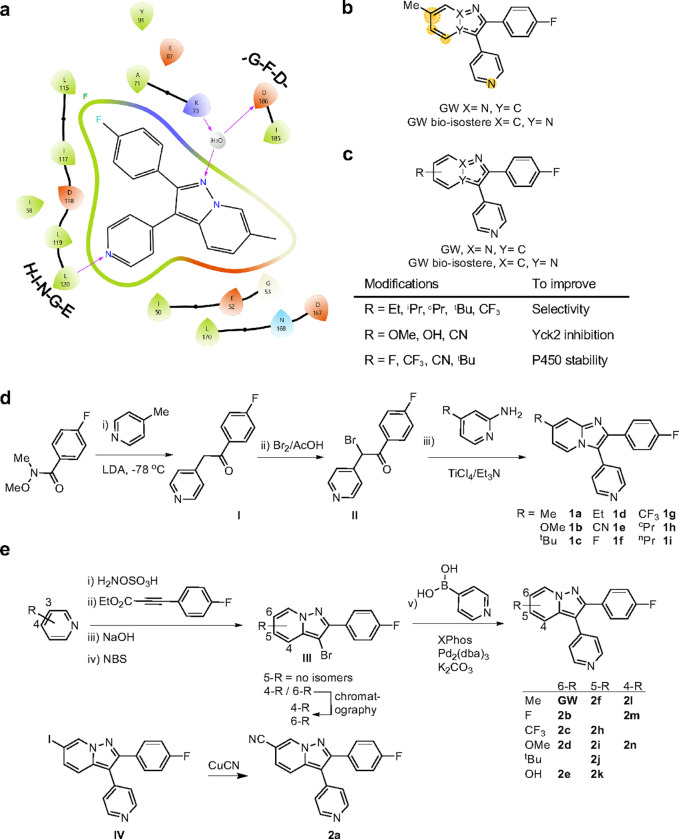
Design and synthesis of GW and its bio-isosters. **a)** Interaction map of GW-Yck2 (PDB: 6U6A) generated with Schrödinger software.^12^ **b)** Identification of metabolism hotspots using SMARTCyp. Yellow highlights indicate regions with the highest predicted susceptibility to oxidation by CYP450 enzymes, with circle size proportionate to the liability score. **c**) Proposed modifications to i) improve Yck2 selectivity over human isoforms, ii) improve Yck2 inhibition, and iii) improve P450 stability. **d**) Synthesis of GW bio-isostere analogs. **e)** Synthesis of GW analogs.

Design and synthesis of new Yck2 inhibitors was performed to overcome the metabolic liabilities of the GW parent scaffold. Initially, design efforts were guided by the co-structure of GW with Yck2 (Fig. 1a, PDB: 6U6A).^12^ Within the ATP-binding pocket, the nitrogen atom of the 4-pyridyl group of GW appeared responsible for a key interaction with the backbone amide of residue L120 in the hinge region of the Yck2 kinase domain, while the 4-fluoro substituent on the 2-phenyl group formed a hydrophobic interaction with L115 (Fig. 1a). Hence, we reasoned that modification of the 4-pyridyl or 2-fluorobenzene substituents could potentially jeopardize kinase inhibition and antifungal activity. In contrast, the pyrazolo[1,5-a]pyridine core of GW extended outwards from the ATP binding pocket into solvent and we reasoned that this area was more likely to tolerate modification without compromising bioactivity. The 6-methyl group on the GW core appeared important for fungal selectivity, but analysis by the SMARTCyp metabolic hotspot predictor indicated that it was also a potential site of hepatic cytochrome P450 oxidation (Fig. 1b).^17^ Therefore, we targeted modifications to the pyrazolo[1,5-a]pyridine core (GW series) and the bio-isosteric imidazolo[1,2-a]pyridine core (GW bio-isostere series) to improve metabolic stability while maintaining kinase selectivity and antifungal activity (Fig. 1c). Ethyl, isopropyl, cyclopropyl, tertiary butyl, and trifluoromethyl modifications on the benzene ring were designed to interact with the hydrophobic region of the P-loop to optimize Yck2 fungal kinase selectivity over the human CK1 isozymes (Fig. 1a). 6-Methoxy, hydroxy, and cyano substituents were selected to modify the polarity of the substituent to increase the potency of Yck2 enzyme inhibition by potentially engaging the carbonyl residues of the DFG loop (residues 186–188). Analogs with 6-fluoro, trifluoromethyl, cyano, and tertiary butyl substituents were predicted by SMARTCyp to improve metabolic stability by reducing cytochrome P450 oxidation.^17^

Initially, analogs were synthesized in the bio-isosteric imidazole[1,2-a]pyridine series due to the greater synthetic accessibility of the core using a three-step procedure (Fig. 1d). The lithiation of methylpyridine followed by a reaction with a Weinreb amide afforded a methyl ketone (Fig. 1d),^18–20^ which upon reaction with bromine in acetic acid provided an α-bromoketone. Subsequently, the GW bio-isostere core was constructed by TiCl_4_-assisted condensation^21^ of the α-bromoketone with a substituted amino pyridine, which allowed diversification of analog preparation in the final step of the synthesis (Fig. 1d).

In contrast to the bio-isoteric imidazole[1,2-a]pyridine series, synthesis of the pyrazolo[1,5-a]pyridine core in the GW series was complicated by the formation of regioisomers during the cyclization reaction (Fig. 1e). The synthesis commenced with a 3-substituted pyridine. *N*-amination of the pyridine followed by cycloaddition with ethyl 3-(4-fluorophenyl) propiolate resulting in a 1:2 to 1:3 mixture of regioisomeric 4- and 6-substituted pyrazolo[1,5-a]pyridines, respectively. Separation of the pyrazolo[1,5-a]pyridines regioisomers was achieved through preparative HPLC to give the pure 4- and 6-substituted analogs. Each intermediate was subjected to a sequence involving hydrolysis, decarboxylation, bromination, and Suzuki coupling, ultimately yielding two series of GW analogs (Fig. 1e).^12^

### Heterocycle-substituted GW analogs retain selective Yck2 inhibitory activity

Following the synthesis of nine GW bio-isostere analogs based on the imidazo[1,2-a]pyridine core and thirteen structure-guided R-substituent analogs of the parent GW pyrazolo[1,5-a]pyridine core, we examined the ability of each analog to inhibit the kinase activity of purified recombinant *C. albicans* Yck2 kinase domain. Purified *C. albicans* Yck2 kinase domain was incubated with ATP (at its Km of 20 μM) and each molecule of interest in a two-fold dilution series up to 1 μM. Relative ADP production in the presence of casein peptide as a substrate was measured (**Table 1**).^12^ The 50% inhibitory concentration (IC_50_) for each molecule was computed and compared to the GW parent and DMSO solvent controls (**Table 1**). We confirmed that 18 of 22 compounds inhibited the kinase activity of purified *C. albicans* Yck2, with an IC_50_ < 1 μM (**Table 1**). Four molecules demonstrated Yck2 IC_50_ values above the maximum concentration tested, **2c** (6-CF_3_), **2h** (5-CF_3_), **2l** (4-Me), and **2o** (4-CN), indicating that these modifications are poorly tolerated.

To evaluate the fungal selectivity of each molecule, we also performed kinase inhibition assays against recombinant human casein kinase I alpha, CK1α. Each molecule of interest was added to CK1α reaction mixtures in a two-fold dilution series from 0–15 μM (**Table 1**), and IC_50_ values for each molecule were computed and compared to GW and DMSO solvent controls (**Table 1**). Where an IC_50_ could be computed for each kinase, *C. albicans* Yck2 was inhibited more potently than *Homo sapiens* CK1α, with selectivity values ranging from 2–41-fold (**Table 1**). Molecules of the imidazo[1,2-a]pyridine bio-isostere group were generally less selective for *C. albicans* Yck2 compared to their pyrazolo[1,5-a]pyridine counterparts, with the average fungal selectivity of bio-isosteres approximately 7-fold compared to approximately 23-fold for the pyrazolopyridine group (**Table 1**). Thus, the greater ease of synthesizing bio-isosteric analogs appears to come at the cost of reduced selectivity for Yck2 in comparison to substituted analogs of the parent pyrazolo[1,5-a]pyridine core.

### Growth inhibition and microsomal stability assays revealed four analogs with antifungal activity and improved metabolic stability

Having achieved promising potency and fungal selectivity for several compounds at the level of fungal target engagement, we next sought to determine which GW analogs retained whole cell antifungal activity. To do so, we performed two-fold dose-response assays with *C. albicans* grown in RPMI medium at 37 °C under 5% CO_2_, conditions that mimic host conditions under which Yck2 is required for growth (**Fig. S1a, Table 1**).^12^ As expected for molecules with Yck2 IC_50_ values > 1 μM (**2c** (6-CF_3_), **2h** (5-CF_3_), **2l** (4-Me), and **2o** (4-CN)), little to no whole-cell antifungal activity was observed. For biochemically potent compounds, we found that substitution of the GW core heterocycle at the 5-position generally resulted in a marked loss of bioactivity, increasing the MIC_80_ of the respective analog relative to GW (**Fig. S1a**, grey labelling; **Table 1**). In contrast, substituting the pyrazolopyridine molecules at the 6- and 4-positions had variable effects on whole cell antifungal activity (**Fig. S1a, Table 1**). Specifically, for both the 6-substituted pyrazolopyridine and imidazopyridine series, several analogs displayed robust anti-*C. albicans* bioactivity, including those with 6-cyano (**1e, 2a**), 6-fluoro (**1f, 2b**), and 6-methoxy (**2d, 1b**) substituents (**Fig. S1a, Table 1**). Additionally, 6-ethyl (**1d**) and 6-cyclopropyl (**1h**) modifications of the imidazopyridine core heterocycle retained bioactivity at 50 μM (**Fig. S1a, Table 1**). Conversely, 6-trifluoromethyl modifications (**2c** and **1g**) resulted in loss of bioactivity. Finally, the 4-fluoro and 4-methoxy substitutions of **2m** and **2n** resulted in MIC_80_ values of 25 μM. We also noted that the imidazopyridine GW bio-isosteres demonstrated MIC_80_ values 2–4-fold higher than those of their pyrazolopyridine counterparts, suggesting that changes to the GW core heterocycle yields molecules with slightly poorer target engagement and/or intracellular accumulation (**Fig. S1a, Table 1**). In fact, only **2a** and **2d** had MIC_80_ values of 12.5 μM, a value comparable to the GW parent scaffold. Furthermore, biochemical inhibition of Yck2 did not always correlate with whole-cell antifungal activity. For example, despite hydroxy-substituted molecules **2e** (6-OH) and **2k** (5-OH) having no measurable whole-cell activity against *C. albicans*, both molecules demonstrated Yck2 IC_50_ values of 100 nM and 110 nM respectively, which were comparable to the GW parent (**Fig. S1a, Table 1**). Such a disconnect suggests that certain substitutions impair intracellular accumulation of the GW scaffold, perhaps reducing the ability of molecules to traverse the fungal cell wall and/or membrane or increasing their efflux out of the cell.

To prioritize molecules for further investigation, we determined whether any of the modifications made to the GW scaffold ameliorated the poor *in vitro* metabolic stability of the parent.^12^ As mentioned previously, the 6-methyl group of the GW parent was predicted to be a hotspot for CYP450 metabolism using the SMARTCyp 3.0 server^17^, prompting an exploration of its modification to improve metabolic stability of analogs. Standard mouse liver microsomal stability assays were performed to predict susceptibility to hepatic Phase I metabolism (**Table 1**). Molecules were broadly categorized as metabolically ‘unstable’ if compound concentration remaining in the microsomal suspension after incubation for 30 minutes was ≤ 50% of the starting concentration (1 μM) and metabolically ‘stable’ if the percent compound remaining was > 50% (**Fig. S1b, Table 1**). As expected, the parent GW, and other methyl-substituted analogs (**1a, 2f, 2l**), were highly unstable (**Fig. S1b, Table 1**). While a majority of GW substituted analogs and bio-isosteres were also highly metabolized, molecules with fluoro-, trifluoromethyl-, and cyano- substituents had markedly improved microsomal stability (**Fig. S1b, Table 1**). Of the metabolically ‘stable’ molecules, two 6-substituted pyrazolo[1,5-a]pyridines (**2a** (6-CN) and **2b** (6-F)) and two 6-substituted imidazo[1,2-a]pyridine bio-isosteres (**1e** (6-CN) and **1f** (6-F)) retained sufficiently promising potency at target and whole-cell bioactivity against *C. albicans* to be prioritized for further characterization (**Fig. S1** and **Table 1**).

### Prioritized molecules 2a, 2b, 1e, and 1f demonstrate potent and selective inhibition of C. albicans Yck2

**Figure 2:**
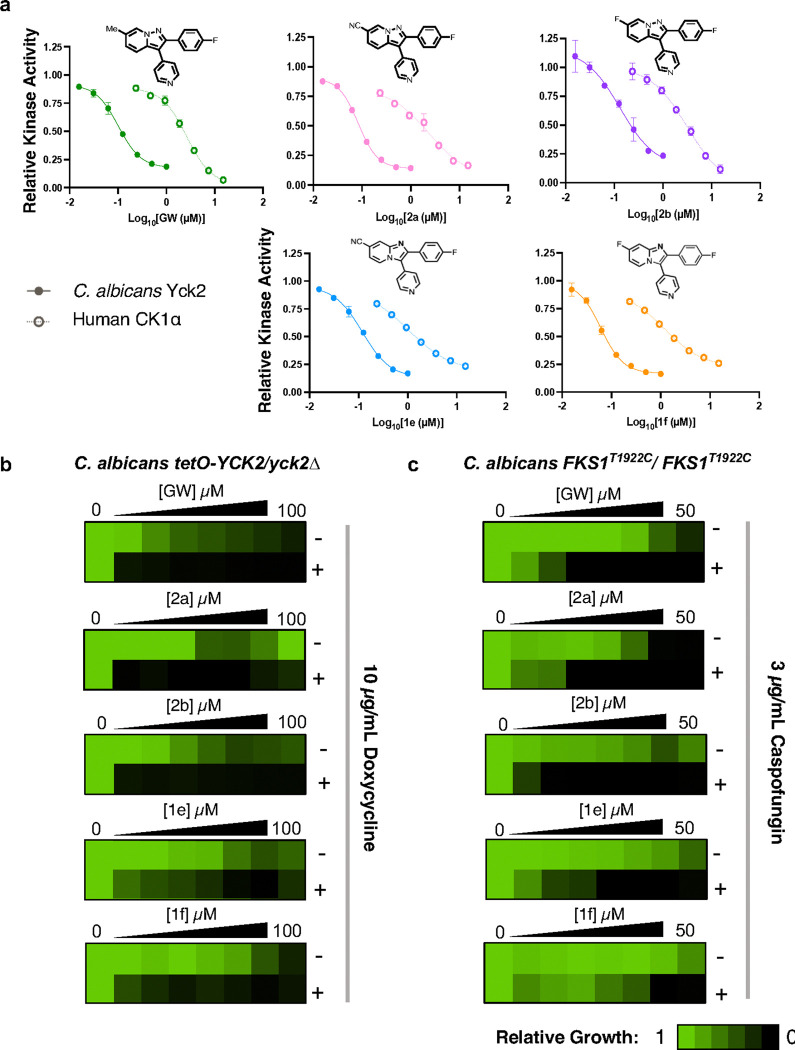
Prioritized Yck2 inhibitors selectively inhibit *C. albicans* Yck2. **a)** Purified *C. albicans* Yck2 kinase domain (0.115 μg/reaction) and human CK1α (0.05 μg/reaction) were treated in two-fold dose response with each Yck2 inhibitor. Reactions were supplemented with dephosphorylated casein as substrate (2 μg) and ATP at the Km of the relevant kinase (*Ca*Yck2: 20 μM, *Hs*CK1α: 10 μM). Following incubation, relative kinase activity was measured using the ADP-Glo^™^ (Promega) bioluminescent assay (100 ms integration). Data were normalized to drug-free controls and IC_50_ values computed using GraphPad Prism 9, *n* = 3, Error bars: ± standard deviation (SD). **b)** The susceptibility of a doxycycline-repressible *tetO-YCK2/yck2Δ* strain of *C. albicans* was evaluated in the presence and absence of doxycycline (10 μg/mL). Two-fold dose-response assays were performed in RPMI at 37 °C under 5% CO_2_ for 48 hours. Growth was determined by optical density at 600 nm (OD_600_) and normalized to drug-free controls (see colour bar). **c)** The ability of each GW analog to potentiate caspofungin was tested in an echinocandin-resistant *C. albicans* strain (DPL15; *FKS1*^*T1922C*^*/FKS1*^*T1922C*^) using a two-fold dose-response assay in YPD at 30 °C for 48 hours in the presence or absence of a sub-inhibitory concentration of caspofungin (3 μg/mL). Growth was determined by optical density at 600 nm (OD_600_) and normalized to drug-free controls (see colour bar).

To further exemplify the patterns of kinase inhibition exhibited by **2a, 2b, 1e**, and **1f**, we compared fungal-selectivity of each inhibitor alongside GW in our biochemical kinase assays. Specifically, we demonstrated that all four analogs inhibited Yck2 with IC_50_ values between 80–130 nM (**Table 1**, Fig. 2a). Compound **2a** demonstrated the highest potency against Yck2 (IC_50_ = ~ 80 nM), which correlated with it being the most bioactive analog against *C. albicans* (MIC_80_ = 12.5 μM). When comparing each analog’s selectivity for Yck2 over human CK1α, the pyrazolopyridine analogs, **2a** (6-CN) and **2b** (6-F), were 25–fold and 21–fold more selective, respectively, values greater than the parent GW (11–fold selectivity; **Table 1**, Fig. 2a). Imidazopyridine bioisosteres **1e** (6-CN) and **1f** (6-F), while not as fungal-selective as the pyrazolopyridine analogs, demonstrated similar fungal selectivity to GW, at 8–fold and 12–fold, respectively (**Table 1**, Fig. 2a).

Moving forward, we sought to obtain confirmation for on-target inhibition of Yck2 by prioritized compounds using a genetic approach based on the principle that a reduction in dosage of the gene encoding a compound’s target kinase will result in hypersensitivity to the compound. We used a *C. albicans* strain in which one allele of *YCK2* is deleted while the other allele is under the control of a doxycycline (DOX)-repressible promoter (*tetO-YCK2/yck2Δ*).^22^ To determine whether reducing the level of *YCK2* in *C. albicans* resulted in hypersensitivity to GW analogs, we performed two-fold dose-response assays with each prioritized molecule in the presence or absence of 10 μg/mL DOX (Fig. 2b). Marked reductions in the MIC_80_ of each molecule in the presence of DOX compared to in the absence of DOX were observed, consistent with GW and its analogs inhibiting Yck2 as their mechanism of antifungal activity (Fig. 2b). Additionally, previous work established that inhibition of Yck2 results in sensitization to cell wall perturbing agents, including echinocandins such as caspofungin.^12,16^ To determine whether this phenotype would occur upon treatment with our prioritized Yck2 inhibitors, we used an echinocandin-resistant *C. albicans* strain (*FKS1*^*T1922C*^*/FKS1*^*T1922C*^), and incubated it with a two-fold gradient of GW, **2a, 2b, 1e**, and **1f** in the presence or absence of a sub-inhibitory concentration of caspofungin. When *C. albicans FKS1*^*T1922C*^*/FKS1*^*T1922C*^ was treated with caspofungin, cells were sensitized to GW, **2a, 2b, 1e**, and **1f** (Fig. 2c). Specifically, reductions in MIC_80_ upon addition of caspofungin reached ≥ 32-fold for GW, **2a, 2b** and **1e**, and ≥ 4-fold for **1f** (Fig. 2c).

### Prioritized Yck2 inhibitors show efficacy against C. albicans when co-cultured with human cell lines in vitro

**Figure 3:**
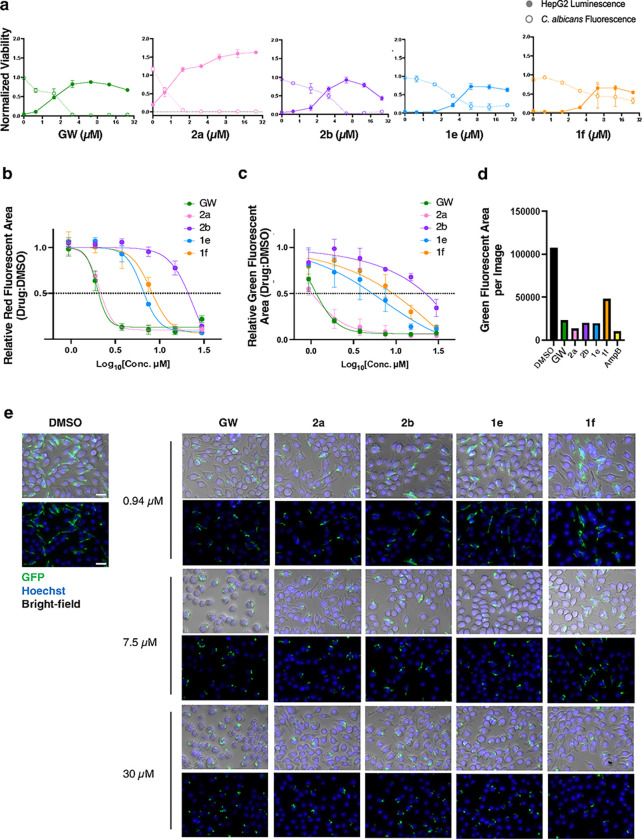
Yck2 inhibitors rescue human cells in a co-culture model of *C. albicans* infection. **a**) Assays were performed to determine if GW and analogs could clear *C. albicans* (*FKS1*^*T1922C*^*/FKS1*^*T1922C*^ Eno1-GFP) from co-cultured luciferized human HepG2 (fLuc CMV) cells grown in RPMI (no phenol red) with 10% HI-FBS at 37 °C under 5% CO_2_. Each compound was added in two-fold dose-response format and after 48 hr incubation, relative fungal burden was determined by GFP fluorescence (Ex485/Em518) while HepG2 growth/survival was determined by luminescence (100 ms integration). Data were normalized to the relevant drug-free mono-cultured controls. Error bars: ± SD, n = 3. **b)** Co-cultures were tested to determine if GW and analogs could rescue *C. albicans*-induced death of phagocytes. Mouse J774A.1 cells were infected with *C. albicans* (MOI: 2) and treated with the indicated concentrations of each compound. Co-cultures were incubated in RPMI with 3% heat-inactivated HI-FBS and 2 μg/mL propidium iodide (PI) at 37 °C under 5% CO_2_ for 24 hours. PI area was quantified using the IncuCyte Basic Analyzer. Data was normalized to DMSO controls and IC_50_ values were computed using GraphPad Prism 10, n = 3. Error bars: ± SD. **c)** J774A.1 cells were infected with *C. albicans* (MOI: 2) that constitutively expresses GFP (*TEF1p*-GFP). After addition of Yck2 inhibitors, co-cultures were incubated in RPMI with 3% HI- FBS at 37 °C under 5% CO_2_ for 16 hours. Green area was quantified using the IncuCyte Basic Analyzer. Data was normalized to DMSO controls, n = 3, Error bars: ± SD **d)** The effects of compounds on phagocytosed *C. albicans* was determined by adding each inhibitor (30 μM) to co-cultures at 1-hour post-infection. Amphotericin B (AmpB, 2 μg/mL) was used as a positive control. Co-cultures were incubated in RPMI supplemented with 3% HI-FBS at 37°C under 5% CO_2_ for 16 hours. Green area was quantified using the IncuCyte Basic Analyzer. The average green fluorescent area from 6 images encompassing technical triplicates from biological duplicates is presented. **e)** To visualize intracellular *C. albicans* (*TEF1p*-GFP) in co-culture with J774A.1 cells, co-cultures were supplemented with the indicated concentrations of each compound and incubated in RPMI with 3% HI-FBS at 37 °C under 5% CO_2_. Images were taken using an AxioVision inverted microscope (Zeiss) at 4-hour post-infection. Hoechst dye was used to stain macrophage nuclei. Macrophage nuclei were visualized as blue signal and *C. albicans* (*TEF1p*-GFP) was visualized as green signal. Scale bar: 1 mm.

We next aimed to elucidate the therapeutic potential of each Yck2 inhibitor *in vitro* using mammalian cell culture. Initially, we performed co-culture experiments using luciferase-labelled HepG2 cells (HepG2-fLuc) and GFP-tagged echinocandin-resistant *C. albicans* (*FKS1*^*T1922C*^*/FKS1*^*T1922C*^ Eno1-GFP) grown together (Fig. 3a) to determine whether each molecule was capable of clearing mammalian cells of the fungus. Co-cultures were treated with a two-fold dose-response series of GW, **2a, 2b, 1e**, or **1f**, and relative viability of each cell type was quantified as a ratio of GFP fluorescence or luminescence measured in compound-treated wells relative to untreated wells. Treatment with GW or the pyrazolopyridine substituted analogs **2a** (6-CN) and **2b** (6-F) effectively cleared *C. albicans* from co-culture and allowed for HepG2 survival at 2.5 μM, 1.25 μM, and 6.25 μM, respectively (Fig. 3a). Notably, **2b** also resulted in a mild reduction in HepG2 viability at concentrations > 12.5 μM, which may indicate off-target toxicity in this human cancer cell line, given the fungal selectivity observed by this molecule during biochemical characterization (**Table 1**). **1e** (6-CN) was also effective at clearing the majority of co-cultured *C. albicans* following treatment at 6.25 μM, while **1f** (6-F) reduced *C. albicans* in co-culture to ~ 50% at or above 6.25 μM relative to compound-free controls, and partially rescued HepG2 viability (Fig. 3a).

To investigate the potential of our Yck2 inhibitors to render *C. albicans* more susceptible to host-mediated immune control, we performed co-culture assays with mouse monocyte-macrophage lineage J774A.1 cells and *C. albicans* (SC5314). The innate immune system is the first line of defense against systemic fungal infections,^23–25^ and macrophages are key effector cells within the innate immune system responsible for phagocytosis and fungal clearance. As a defence mechanism, *C. albicans* undergoes cell wall remodeling and morphogenesis upon phagocytosis by macrophages, which results in macrophage death and promotes fungal escape.^23–27^ Given that Yck2 acts to maintain cell wall integrity and support the morphogenesis of *C. albicans*, we reasoned that Yck2 inhibition might improve the ability of phagocytes to survive and clear fungal infection in co-cultures.^16^ To test this hypothesis, J774A.1 cells and *C. albicans* (SC5314) were co-incubated in the presence of the membrane-impermeable dye propidium iodide (PI) to quantify macrophage cell death (Fig. 3b). Treatment with GW, **2a, 2b, 1e**, and **1f** demonstrated dose-dependent rescue of macrophage viability, with treatment of 30 μM for each molecule restoring full viability in co-culture (Fig. 3b). Specifically, GW, **2a**, and **1e** were most effective in rescuing macrophage viability with IC_50_ values of 2.05 μM and 2.17 μM, respectively (Fig. 3b).

To elucidate the mechanism underlying restoration of macrophage viability in co-culture, we examined fungal growth and intracellular hyphal formation of *C. albicans* treated with the prioritized Yck2 inhibitors.^23,24,27^ To quantify fungal growth in co-culture, we infected macrophages with a strain of *C. albicans* that constitutively expresses GFP (*TEF1p-GFP*) and measured the area of green signal at 16-hours post-infection. We observed that GW and **2a** were the most effective treatments in reducing *C. albicans* growth in co-culture (Fig. 3c). Furthermore, we found that all Yck2 inhibitors reduced *C. albicans’* growth in macrophages at a concentration of 30 μM (Fig. 3d). Finally, to determine whether the Yck2 inhibitors reduce phagocyte death by inhibiting the *C. albicans* yeast-to-filament transition, we examined the morphology of *C. albicans* in co-culture after a 4-hour incubation, a time at which most *C. albicans* cells are internalized and begin to transition to filamentous growth within the phagolysosome.^27^ Treatment with GW, **2a, 2b, 1e**, and **1f** inhibited filamentation of *C. albicans* in a dose-dependent manner, locking *C. albicans* in its yeast form (Fig. 3e). Collectively, these data demonstrated that the Yck2 inhibitors rescue macrophage viability in co-culture by inhibiting fungal growth and filamentation.

### 2a and 1e occupy a similar binding site to GW in C. albicans Yck2

**Figure 4:**
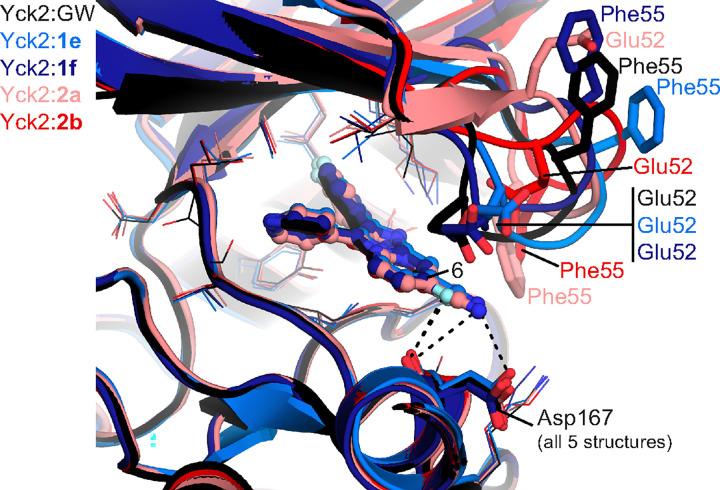
Structural insights into binding mode of Yck2 inhibitors with GW. Structure of the Yck2:GW (black), Yck2:1e (blue), and Yck2:2a (pink) complexes. Protein shown in cartoon representation and molecules shown in ball-and-stick. Key residues as well as the 6-C position of the core heterocycle of the compounds are marked with text.

To gain further insight into the structural basis of Yck2 inhibition, we resolved co- structures 1e, 1f, 2a, and 2b in complex with Yck2 (see **Table S1** for crystallographic statistics). The compounds occupied a nearly identical position in the Yck2 ATP binding site as GW (PDB 6U6A^12^), with the 6-position of the pyrazolopyrimidine core positioned outwards from the core of the pocket towards the solvent accessible region (Fig. 4). The 6-CN groups of **1e** and **2a** extended closer to Asp167, a highly conserved residue among CK1 orthologs, forming a favourable N-O polar interaction with this residue, an interaction that the 6-Me group of GW could not form. All four compounds also interacted with the backbone carbonyl of Asp167 via their 6-F or 6-CN groups, representing another interaction not possible with GW. Interestingly, the structures revealed that the Yck2 P-loop in the **1e**- and **1f**-bound structures adopted a conformation most similar to the GW bound structure, with the Glu52 residue approaching 4 to 5 Å of the 6-group and the Phe55 residue positioned > 10 Å from the compounds. Binding of **2a** or **2b** induced positioning of the P-loop such that the Phe55 residue approached closer to the compounds, within 4 to 6 Å, but only **2b** induced positioning of the Glu52 residue < 6 Å to the 6-group (Fig. 4). These observations suggest that interactions between these compounds and the Yck2 P-loop could be transient, with potential stabilizing interactions between Glu52, Phe55, and the 6-substituent. Overall, however, each of **1e, 1f, 2a**, and **2b** formed additional interactions with Yck2 compared to GW.

### 1e and 2a show the most favourable pharmacology in mice

Given the promising findings observed in co-culture experiments, we proceeded to examine the *in vivo* pharmacology of each analog to determine whether any would be suitable for antifungal efficacy studies in mice and if so, what would be appropriate dosing parameters. We first collected snapshot plasma pharmacokinetic (PK) profiles over 6 hours in mice dosed with GW, **1e**, **1f**, **2a**, or **2b** either orally (p.o.) at 25 mg/kg or intravenously (i.v.) at 5 mg/kg (**Table S2, Fig. S2**). Overall, the PK profiles of our prioritized analogs were greatly improved compared to GW. Importantly for an antimicrobial, the analogs demonstrated good oral bioavailability and long terminal half-lives (> 10 hours) after p.o. administration (**Table S2, Fig. S2**). The 6-cyano substituted molecules (**2a** and **1e**) demonstrated the most promising PK profiles with extended half-lives, and maximal plasma concentrations in the range of effective concentrations *in vitro*. After 25 mg/kg p.o., the maximal plasma concentration (C_max_) of **2a** was ~ 15 μM, which is greater than its MIC_80_ in standard dose-response assay (**Fig. S1a, Table 1, Table S2**), and > 11-fold the concentration required to clear *C. albicans* (*FKS1*^*T1922C*^*/FKS1*^*T1922C*^ Eno1-GFP) co-cultured with HepG2 cells (Fig. 3a). Reflecting its greater oral bioavailability, the C_max_ of **1e** was ~ 36 μM, which is greater than the MIC_50_ of this molecule (Fig. 2b, **Table 1, Table S2**) and > 7-fold the concentration required to clear *C. albicans* (*FKS1*^*T1922C*^*/FKS1*^*T1922C*^ Eno1-GFP) co-cultured with HepG2 cells (Fig. 3a). Dosing of analogs at 5 mg/kg IV did not provide adequate compound exposure, particularly of **1e** and **2a**, which did not reach concentrations consistent with effective antifungal concentrations *in vitro* (**Table S2, Fig. S2b**). Additionally, clearance was more rapid after i.v. administration (**Table S2, Fig. S2b**), leading us to prioritize the oral route of administration for further studies.

**Figure 5:**
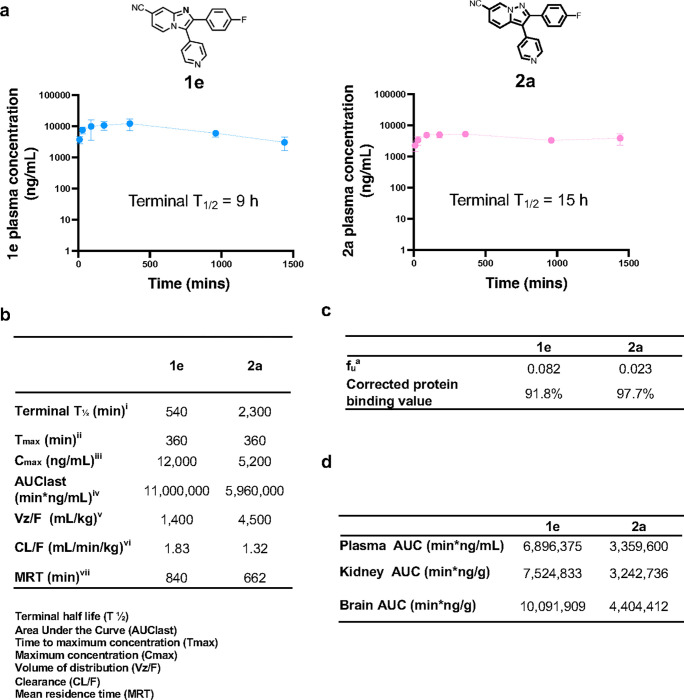
Cyano-substituted compounds 1e and 2a have desirable pharmacology. **a)** Female CD1 mice (n = 3) were dosed (25 mg/kg PO) with **1e** (blue) or **2a** (pink). At the indicated times post-dose, plasma was collected and compound concentrations determined by LC-MS/MS; Mean +/− SD is plotted. **b)** A sparse non-compartmental (NCA) model was used in Phoenix 64 WinNonlin 8.3.3.33 to calculate standard PK parameters. **c)** The extent of plasma protein binding by **2a** or **1e** (5 μM) was determined by rapid equilibrium dialysis. Binding of each compound was determined in triplicate samples using a 6-hr incubation period; fu^a^ = fraction unbound. **d)** Female CD1 mice (n = 3) were dosed with **2a** or **1e** (50 mg/kg PO). At 30, 90 and 360 mins post-dose, samples were collected and LC-MS/MS used to measure compound levels. Tissue concentrations were corrected for contamination by blood in the vasculature of the relevant organ using literature values for the volume of blood in brain (0.03 mL/g) and kidney (0.34 mL/g).

Collectively, the results of our 6-hour snapshot PK profiling experiments motivated us to prioritize **1e** and **2a** for full 24-hour PK profiling studies. **1e** or **2a** were administered once orally at 25 mg/kg and samples were collected over 24 hours. **1e** and **2a** persisted in mouse plasma over the entire 24-hour period with t_1/2_ calculated values of 9 and 15 hours, respectively, suggesting that both could be suitable for once or twice daily dosing in an infection model (Fig. 5a and Fig. 5b). C_max_ values for both molecules mirrored those observed in the p.o. snapshot PK profiles (Fig. 5b and **Table S1**). Additionally, when mice were dosed with 25 mg/kg of **1e** or **2a** once daily over 3 days, accumulation of compound was not observed in the plasma and clearance was little changed suggesting minimal induction of metabolism upon repeated dosing. Importantly, no changes in animal weight or behaviour were observed, highlighting that not only do the molecules demonstrate excellent oral bioavailability and plasma PK, but they are also non-toxic in uninfected mice.

To investigate additional properties of **1e** and **2a** that could impact their antifungal activity *in vivo*, we measured plasma protein binding by equilibrium dialysis *in vitro* and penetration of organs targeted by *C. albicans* (brain and kidney) in mice during systemic infection. Although both **1e** and **2a** exhibited relatively high plasma protein binding (91.8% and 97.7%, respectively, Fig. 5c), oral administration of compounds (50 mg/kg) yielded very good exposure in plasma, brain, and kidney (Fig. 5d). Indeed, **1e** and **2a** could not only cross the blood-brain barrier, but also accumulated in brain tissue at levels higher than the systemic circulation.

### Prioritized Yck2 inhibitors 1e and 2a reduce fungal burden in a mouse model of invasive candidiasis

Encouraged by their antifungal activity in culture and favorable pharmacological properties in mice, we proceeded to evaluate the therapeutic potential of **1e** and **2a** in a neutropenic mouse model of drug-resistant candidiasis. These compounds displayed adequate single-agent activity *in vitro*, and the addition of caspofungin dramatically enhanced their efficacy (Fig. 2c). This biology motivated us to move forward with a 4-arm study where we assessed both the single-agent and echinocandin-potentiating activity of the inhibitors. As an initial pilot experiment, neutropenic female CD1 mice were infected intravenously with 1 × 10^6^ CFU/mL of echinocandin-resistant *C. albicans* (DPL15; *FKS1*^*T1922C*^*/FKS1*^*T1922C*^) and treated one daily for a total of 4 doses with vehicle controls, **1e** alone (p.o.: 25 mg/kg), a non-curative concentration of caspofungin alone (i.p.: 2 mg/kg), or the combination of **1e** and caspofungin (**Fig. S3**). The effects of treatments on fungal burden were evaluated by measuring colony forming units (CFUs) from kidneys resected 24 hours after the final dose of test materials. **1e** alone at the dose and schedule used did not eradicate *C. albicans*, but reduced the fungal burden in the kidneys by approximately 1-log_10_ (**Fig. S3**). Caspofungin alone had a similar effect, while the combination of **1e** with caspofungin resulted in a greater reduction (~ 2-log_10_) in kidney fungal burden compared to either treatment alone (**Fig. S3**). Importantly, no signs of toxicity from any compound were observed, suggesting higher concentrations of both caspofungin and Yck2 inhibitor could be used.

**Figure 6:**
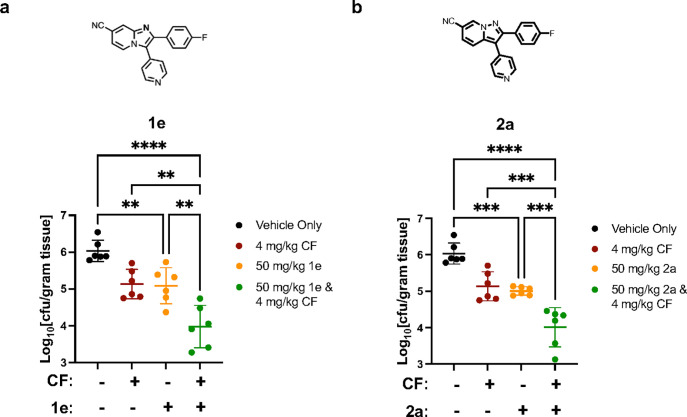
Brief treatment course with 1e and 2a reduces fungal burden in a mouse model of drug-resistant systemic candidiasis. Neutropenic female CD1 mice were infected with *C. albicans* DPL15 (*FKS1*^*T1922C*^*/FKS1*^*T1922C*^) by lateral tail vein injection. Mice were dosed twice daily (50 mg/kg PO) for a total of four doses with either **a) 1e** or **b**) **2a**. In other treatment groups, high dose caspofungin (4 mg/kg IP) once daily for a total of two doses, or a combination of **2a** or **1e** with caspofungin was given as indicated. Control groups received equal volumes of vehicles alone on the same schedule as the combination groups. Kidneys were resected 12 hours after the final dose of test materials. Fungal burden from control and treatment groups is plotted as CFU per gram wet kidney weight (*n* = 6 mice/treatment group; Error bars: ± SD). Statistical significance was determined by one-way ANOVA with SIDAK’s multiple comparison adjustment. **** *p*<0.0001, *** *p*<0.001, ** *p*<0.002.

Encouraged by the initial results, we followed up by infecting CD1 mice once more with echinocandin-resistant *C. albicans* and treating animals with vehicle alone, **1e** or **2a** at an intensified dose and schedule (p.o: 50 mg/kg twice daily, 4 doses in total), caspofungin (i.p.: 4 mg/kg once daily, 2 doses in total), or the combination. Fungal burden was once again evaluated by obtaining CFU counts from kidneys resected 12 hours after the final dose of test materials. Both Yck2 inhibitors as single agents demonstrated significant reductions in kidney CFU relative to vehicle controls (Fig. 6). Moreover, the combination of caspofungin with **1e** or **2a** resulted in significant reduction in fungal burden relative to individual drug treatments, with a greater than 2 log_10_ reduction in CFU relative to vehicle control group (Fig. 6). While a molecule with more potent baseline fungal bioactivity and/or more favourable PK properties is likely required to demonstrate a more significant reduction in kidney fungal burden, this work presents the first evidence that targeting Yck2 shows therapeutic efficacy *in vivo*.

## Discussion

As fungal pathogens pose an ever increasing threat to human health, there remains a great need to expand the current antifungal arsenal. To begin addressing this unmet need, we now describe a multidisciplinary hit-to-lead drug development effort integrating medicinal chemistry, structural biology, biochemistry, microbiology, and pharmacology to demonstrate that targeting the stress kinase Yck2 in *C. albicans* provides a promising therapeutic strategy. Starting from a previously reported phenotypic screen hit, the 2,3-aryl-pyrazolopyridine Yck2 inhibitor GW, we used structural insights to guide synthesis of metabolically stable Yck2 inhibitors with attractive, much improved pharmacology and *in vivo* antifungal activity. While further development of these leads to generate compounds with sub-micromolar antifungal activity will likely be required to generate a clinical candidate, the work presented here provides proof-of-concept that small molecule inhibitors of the kinase Yck2 can exert single-agent activity in mice infected with drug-resistant *C. albicans* and combine with a conventional echinocandin to further improve disease control.

Although *C. albicans* is the focus of the studies presented here, our previous work has shown that GW itself possesses antifungal activity against other critically-important fungal pathogens, including *C. auris* and the basidiomycete *Cryptococcus neoformans*.^12^ Interestingly, no single-agent antifungal activity was observed *in vitro* against *Nakaseomyces glabratus* (formerly referred to as *Candida glabrata*) or *Aspergillus fumigatus*.^12^ Whether this is due to lack of target engagement, lack if intracellular compound accumulation, or divergent biology for Yck2 in these species remains to be determined. Developing a broader understanding of the role of Yck2 homologs in the biology of organisms with diminished susceptibility to GW and related molecules would support the validation of casein kinase 1 inhibition as a viable broad-spectrum antifungal strategy. A potential explanation the lack of effect of Yck2 inhibitors on *N. glabratus* growth is the close evolutionary relationship of this species to *Saccharomyces cerevisiae*, which encodes an additional member of the casein kinase I family, Yck1. Functional redundancy between Yck1 and Yck2 may require both kinases to be impaired for a reduction in cellular viability.^28^ Complete functional characterization of *A. fumigatus* casein kinases has yet to be completed, however, studies in *Aspergillus nidulans* determined that the CK1 homolog, CkiA, is essential for survival, suggesting that druggable candidate casein kinases are present in the *Aspergillus* kinome.^29^ Future work will be needed to interrogate whether functional or structural divergence in the target of GW analogs is sufficient to explain their species-restricted inhibitory activity observed for the inhibitors characterized to date. Should this phenomenon result from physical barriers impairing compound accumulation within the fungal cell of increased efflux in some species, additional structural optimization remains a possibility to further develop pyrazolo[1,5-a]pyridine and/or imidazo[1,2-a]pyridine core molecules with improved activity spectra.

The co-structures defining the binding modes of **1e**, **1f**, **2a** and **2b** within the Yck2 kinase domain revealed that **2a** and **2b** bind to Yck2 with unique P-loop conformations as compared to GW, while **1e** and **1f** induce P-loop conformations similar to that induced by GW binding. Upon the initial structural and functional characterization of GW, it was hypothesized that positioning of fungal-specific residue Glu52 within the P-loop, which adopted a ‘folded’ conformation, was important in facilitating inhibitor binding with the Yck2 kinase domain’s active site.^12^ This may have been achieved with **1e** and **1f**, but overall our observations suggest that the interactions of these four compounds with the P-loop may be transient. To improve Yck2-binding affinity, additional medicinal chemistry efforts could be undertaken to design more strategic 6-substituents of the pyrazolo[1,5-a]pyridine and imidazo[1,2-a]pyridine scaffolds that would stabilize P-loop interactions while taking care to avoid the introduction of any new metabolic liabilities.

Despite the conservation of kinases across the kingdoms of life, these enzymes play critical roles in microbial growth, proliferation, virulence, and the response to xenobiotic stress, making them attractive targets for antimicrobial drug development.^30^ Work in bacteria revealed that inhibitors of histidine kinases and serine/threonine kinases can be useful as single-agents, or as potentiators of other antibiotic classes.^30–32^ Our findings largely mirror those in fungal pathogens, and although no antifungal kinase inhibitors are currently licenced for clinical use, their potential for development as therapeutics remains bright.^12–15^ Studies in *C. albicans* demonstrated that pharmacological inhibition of diverse kinases including TOR^14^ and Pkc1^15,33,34^ results in synergy with the azoles, offering an innovative solution to cope with rising rates of drug resistance. Additionally, inhibition of Pkc1 has been associated with reductions in virulence in mammalian models of fungal infection,^33^ and 1-acetyl-β-carboline (1-ABC), an inhibitor of the dual-specificity tyrosine phosphorylation-regulated kinase (DYRK) Yak1, represses the yeast-to-filament transition in *C. albicans*, a key virulence trait.^35^ As such, this beta-carboline along with similar analogs can block the formation of biofilms *in vitro*, and in a rat catheter model of *C. albicans* infection.^35^ While our work describes efforts to target fungal casein kinases, work in *Leishmania* demonstrated that pharmacological inhibition of casein kinase 1 family members blocks intracellular survival and infectivity of the parasite.^36,37^ These studies also revealed that the ATP-binding domain of the abundant CK1 isoform *Lm*CK1.2 differs in structure from that of human CK1s,^36,37^ similar to the observations we made comparing *C. albicans* Yck2 with human CK1α. Such divergence suggests that the casein kinase 1 family could provide a particularly good opportunity for structure-guided development of highly selective antimicrobial agents in the future. Finally, our findings help establish protein kinases more broadly as a rich target space for discovery and development of new, mechanistically distinct antifungals. Moreover, due to their activity in combination with a mainstay of the current antifungal armamentarium, the leads we report here have the potential, with further development, to provide the kind of resistance-aversive combination approach to antifungal therapy that has proven key to controlling other systemic infectious diseases.

## Methods

### General synthetic procedure

All reagents and solvents used were purchased from commercial sources and were used without further purification. NMR spectra were obtained using a Bruker 850 MHz (UNC-CH) or Bruker 500 MHz (UNC-CH) or Bruker 400 MHz (UNC-CH) or Bruker 400 MHz (at Piramal Pharma) or INOVA 400 MHz spectrometers at room temperature; chemical shifts are expressed in parts per million (ppm, δ units) and are referenced to the residual protons in the deuterated solvent used. Coupling constants are given in units of hertz (Hz). Splitting patterns describe apparent multiplicities and are designated as s (singlet), d (doublet), t (triplet), q (quartet), m (multiplet), and br s (broad singlet), dd (doublet of doublets), ddd (double double doublet), tt (triplet of triplets). The purity of compounds submitted for biological screening was determined to be ≥ 95% as measured by HPLC. Analytical thin layer chromatography (TLC) was performed on silica gel plates, 200 μm with an F254 indicator. Column chromatography was performed using RediSep Rf^®^ preloaded silica gel cartridges on Isolera one Biotage automated purification systems. Samples for high-resolution mass spectrometry were analyzed with a ThermoFisher Q Exactive HF-X (ThermoFisher, Bremen, Germany) mass spectrometer coupled with a Waters Acquity H-class liquid chromatograph system. Samples were introduced via a heated electrospray source (HESI) at a flow rate of 0.3 mL/min. Electrospray source conditions were set as: spray voltage 3.0 kV, sheath gas (nitrogen) 60 arb, auxillary gas (nitrogen) 20 arb, sweep gas (nitrogen) 0 arb, nebulizer temperature 375 degrees C, capillary temperature 380 ∘ C, RF funnel 45 V. The mass range was set to 150–2000 m/z. All measurements were recorded at a resolution setting of 120,000. Separations were conducted on a Waters Acquity UPLC BEH C18 column (2.1 × 50 mm, 1.7 *μ*m particle size). LC conditions were set at 95% water with 0.1% formic acid (A) ramped linearly over 5.0 mins to 100% acetonitrile with 0.1% formic acid (B) and held until 6.0 mins. At 7.0 mins the gradient was switched back to 95% (A) and allowed to re-equilibrate until 9.0 mins. Injection volume for all samples was 3 *μ* L. Analytical LC/MS data was obtained using a Waters Acquity Ultrahigh-performance liquid chromatography (UPLC) system equipped with a photodiode array (PDA) detector using the following method: solvent A = Water + 0.2% FA, solvent B = ACN + 0.1% FA, flow rate = 1mL/min. The gradient started at 95% A for 0.05 min. Afterwards, it was ramped up to 100% B over 2 min and held for an additional minute at this concentration, before returning to the initial gradient. Compounds were purified on prep HPLC using an Agilent 1100 equipped with a Phenomenex column (Phenyl-Hexyl, 75 × 30 mm, 5 μm) using the following method: Solvent A: water + 0.05% TFA; Solvent B: MeOH; flow rate: 70.00 mL/min. LC conditions were set at 90% (A) ramped linearly over 8.0 mins to 100% (B) and held until 10.0 mins at 100% B. At 10.0 mins the gradient was switched back to 90% (A). Detailed synthesis notes and spectra for all molecules generated for this project can be found in **Supplementary File 2.**

### Fungal strain and culture conditions

Archives of all strains were maintained at −80 °C in 25% glycerol. Strains were grown in standard conditions at 30 °C in YPD (1% yeast extract, 2% peptone, 2% dextrose), unless otherwise indicated in RPMI (10.4 g/L RPMI powder with L-glutamine (Gibco), 165 mM MOPS, 2% glucose, 5 mg/mL histidine, pH 7), or SD (2% glucose, 6.7 g/L yeast nitrogen base without amino acids). All strains used in this study are listed in Table S3.

### Dose-response assays

Drug susceptibility assays were performed in 384-well plates in a final volume of 0.04 mL/well with two-fold dilutions of each compound in YPD or RPMI medium, as indicated. Plates were incubated in the dark at 30 °C or 37 °C under static conditions, and OD600 was measured after the indicated incubation times using a spectrophotometer (Molecular Devices). For assays employing strains from the GRACE (*tetO*) collection, strains were grown overnight in the presence and absence of the indicated doxycycline (DOX) concentration(s). Data were quantitatively displayed as heat maps using Java TreeView3.

### Metabolic stability assays (Analiza Inc.)

Mouse liver microsomal stability assays were completed by Analiza Inc. Compounds were supplied as 10 mM DMSO stocks and diluted serially to 2.5 mM (DMSO) and 0.5 mM (acetonitrile). Reaction plates were prepared with 691.25 μL, pre-warmed (37 °C) mouse liver micosomes (0.63 mg/mL in 100 mM KPO_4_ + 1.3 mM EDTA) to an empty well of a 96 well plate and maintained at 37°C. Test compounds were added to microsomes in the reaction plate and mixed. Solutions were pre-incubated for 5 m at 37°C. Compound at t = 0 was determined by aliquoting a sample of each reaction solution and adding MeOH and NADPH regeneration solution immediately. The remaining reaction solutions were incubated in the presence and absence of NADPH for 30 m at 37 °C before adding MeOH to quench the reactions. LC-TOFMS was used to quantify each test compound. Data acquisition was completed using Agilent 6538 Ultra High Accuracy TOF MS (m/z 100–1000) using generic conditions in positive mode. Exact mass and peak integration was determined using MassHunter (Agilent). Data were expressed as percent remaining of unchanged parent compound at each time point.

### Kinase inhibition assays

Kinase assays were performed using the ADP-Glo kinase assay kit (Promega) in solid white 384 well plates (Corning). Assays with were performed in kinase buffer (1x: 2 mM NaHEPES pH 7.5, 650 mM KCl, 50 mM MgCl_2_, 25 mM b-glycerophosphate) supplemented with 2 μg casein kinase I peptide substrate (SignalChem) per reaction, and ~ 0.115 μg purified recombinant *Ca*Yck2 kinase domain or 0.05 μg purified *Hs*CK1a (Abcam) per reaction as indicated. Each kinase inhibitor of interest was added in a two-fold dilution series at the concentrations indicated, followed by the addition of ATP at 20 μM (*Ca*Yck2 KmATP) or 10 μM (*Hs*CK1a). Assays were performed in 10 μL reactions (*n* = 3) and incubated for 30 minutes at 30°C. ADP-Glo kinase assay reagents (Promega) were applied to assay wells per manufacturer’s instructions. Luminescence was measured with a TECAN Spark^®^ multimode microplate reader, and background luminescence was subtracted from reaction wells from control wells incubated with all reaction reagents except for the relevant kinase enzymes. IC_50_ values were calculated, and data plotted using GraphPad Prism 9’s Nonlinear fit function.

### Co-culture assays

HepG2 cells (ATCC, male, CAT# HB-8065) infected with a lentiviral vector expressing firefly luciferase from a CMV promoter (HepG2-fLuc) were grown in DMEM medium (Gibco) with 10% fetal bovine serum (FBS). Experiments were performed in cells within 12 passages post-recovery from stocks stored in liquid nitrogen and confirmed to be PCR negative for mycoplasma contamination. HepG2-fLuc cells were seeded at 1 × 10^5^ cells/mL in black-walled, clear-bottom 384-well plates. Following overnight incubation at 37 °C with 5% CO_2_ continuous infusion, GFP-labelled *C. albicans* DPL15 (Eno1-GFP; CaLC6194) was added to assay wells seeded with HepG2-fLuc cells at a concentration of 2.5 × 10^4^ cells/mL. Two-fold dilutions of each test compound were added to assay wells at the concentrations specified in text. Control plates containing monocultures of HepG2-fLuc cells or *C. albicans* were prepared using equal compound titrations. All assay plates were incubated for 48 hours at 37 °C with 5% CO_2_. Relative fluorescence of each well was measured using a TECAN Spark^®^ multimode microplate reader at Ex.485/Em.535. Following fluorescence readings, 5 μL Steady-Glo^®^ Luciferase Assay reagent was added to each assay well prior to incubation at room temperature for 10 minutes. Relative luminescence was read using a TECAN Spark^®^ multimode microplate reader. Reported results are representative of two biological replicates, each of which was performed in technical triplicate.

### Macrophage killing assay

To quantify macrophage death in co-culture with *C. albicans*, mouse monocyte-macrophage lineage J774A.1 cells were seeded in a 96-well tissue culture-treated plates by adding 100 μL of cell suspension (4 × 10^5^ cells/mL) into each well. Cell culture was grown in RPMI medium supplemented with 3% HI-FBS for 18 h at 37°C + 5% CO_2_. On the following day, overnight culture of wild-type *C. albicans* (SC5314) was washed threes time with PBS and diluted to 8× 10^5^ cells/mL in RPMI medium supplemented with 3% HI-FBS and 2μg/mL of propidium iodide (PI, Sigma, P417). Infection was performed by adding 100 μL of fungal cell suspension to each well previously seeded with J774A.1 cells. Co-cultures were treated with drugs of interest in a two-fold serial dilution, starting at 30 μM. Equal volume of DMSO to Yck2 inhibitor was used as a vehicle control. Co-cultures were incubated for 24 h at 37°C + 5% CO_2_. Co-cultures were imaged using the IncuCyte^®^ S3 Live-Cell Analysis System. Red area was quantified using the IncuCyte Base Analysis Software. Data was normalized to DMSO controls.

### Quantification of C. albicans growth in co-culture with macrophages

To examine the growth of *C. albicans* co-culture with macrophage in the presence of drug treatment, J774A.1 cells were seeded in a 96-well plate by adding 100 μL of 4 × 10^5^ cells/mL of cell suspension to each wall. Plates were incubated for 18 h at 37°C + 5% CO_2_. On the following day, *C. albicans* (*pTEF1-GFP*) overnight cultures were washed three times with PBS and diluted to 8 × 10^5^ cells/mL in RPMI medium supplemented with 3% HI-FBS. *C. albicans* cell suspension (100 μL/well) was added to the wells previously seeded with J7 cells and incubated for 16 h at 37°C + CO_2_. Co-cultures were treated with each drug of interest in two-fold serial dilutions, starting at 30 μM. Equal volume of DMSO to Yck2 inhibitor was used as a vehicle control. Data was normalized to DMSO controls.

To determine the effects of the Yck2 inhibitors on the growth of intracellular *C. albicans*, J774A.1 cells and phagocytosed *C. albicans* co-cultures were washed with PBS to remove extracellular *C. albicans* and treated with 30 μM of each drug of interest dissolved in RPMI medium supplemented with 3% HI-FBS at 1-hour post-infection. Equal volume of DMSO to Yck2 inhibitor was used as a vehicle control. 2 μg/mL amphotericin B was used as a positive control.^38^ Co-cultures were imaged using an IncuCyte^®^ S3 Live-Cell Analysis System and green area was quantified using the IncuCyte Base Analysis Software.

### Visualization of intracellular C. albicans in macrophages

To monitor *C. albicans* filamentation in J774A.1 cells, J774A.1 cells were seeded in a 96-well plate by adding 100 μL of cell suspension (4 × 10^5^ cells/mL) to each wall. Plates were incubated for 18 h at 37°C + 5% CO_2_. On the next day, overnight *C. albicans* (*pTEF1-GFP*) cultures were washed three times with PBS and diluted to 8 × 10^5^ cells/mL in RPMI medium supplemented with 3% HI-FBS. 100 μL of fungal cell suspension was added to the J774A.1-seeded 96-well plate. Co-cultures were treated with either 0.94 μM, 7.5 μM, or 30μM of GW, YK-I-02, YK-I-03, MN-I-157, or MN-I-158 and incubated in RPMI + 3% HI- FBS at 37°C + 5% CO_2_ for 4 h. Macrophage nuclei was stained with 5 μg/mL of Hoeschst stain (Invitrogen, Hoechst 33342) and visualized via blue signal. *C. albicans* (*pTEF1-GFP*) were visualized via green signal. Images were obtained with an AxioVision inverted microscope (Carl Zeiss) using phase contrast optics, white light illumination, and an X-cite series 120 light source for fluorescence excitation.

### Pharmacokinetic (PK) profiling

PK profiles were generated for GW and prioritized analogs using female CD1 mice (*n* = 3) from Charles River (Wilmington, MA) dosed orally (PO) at 25 mg/kg or intravenously (IV) at 5 mg/kg and monitored for 6 or 24 hours, as indicated. Drug was administered in the following vehicles: 10% DMSO + 90% of 0.5% Methocel A4M/0.2% Tween 20 for P.O. administration and 10% DMSO + 90% of 20% solution of captisol for I.V. administration. At the indicated times post-dose, mice were submandibular bled or overdosed with CO_2_ and bled by cardiac puncture. Plasma was obtained by centrifugation of blood for 10 min at 9,600 × g at 4 °C. When analyzed, tissues were collected, rinsed to remove surface adhering blood, weighed and snap frozen. Upon thawing, tissues were homogenized in 3X weight by volume PBS. Vendor supplied K_2_EDTA plasma from BioIVT (Westbury, NY) or blank tissue homogenate spiked with known concentrations of each compound was used to prepare standards and quality control samples. 100 μL samples or standards were crashed with 300μL of acetonitrile containing formic acid (0.1% in final mixture) and n-benzylbenzamide internal standard (50 ng/mL in final mixture). Tubes were vortexed, incubated at room temperature (RT) for 10 minutes, and spun for 5 minutes at 16,100 × g. Supernatant was transferred to a second tube and again spun for 5 minutes at 16,100 × g. Supernatant was analyzed by LC-MS/MS on a Sciex (Framingham, MA) 4500 Triple Quad^™^ system coupled to a Shimadzu (Columbia, MD) Prominence LC. An Agilent (Santa Clara, CA) C18 XDB column (5 micron packing 50 X 4.6 mm size) was used for chromatography under gradient conditions with water (Buffer A) and methanol (Buffer B) both containing 0.1% formic acid. Compounds were detected in multiple reaction monitoring (MRM) mode using ESI. Transitions monitored were as follows: **GW** 304.057/208.0; **1e** 315.112/185.2; **1f** 308.271/185.2; **2a** 314.787/219.0; **2b** 308.011/212.0.:. Tissue concentrations were determined after subtraction of drug in tissue vasculature using measured plasma concentrations and literature values^39^ or the volume of blood in brain (0.03 mL/g) and kidney (0.34 mL/g). Compounds were assumed to partition equally between plasma and red cells. Phoenix 64 WinNonlin 8.3.3.333 (Certara, Corp, Radnor, PA) was used to calculate PK parameters for half-life, area under the curve (AUC), T_max_, C_max_, clearance, volume of distribution, and mean residence time. A sparse non-compartmental (NCA) model was used.

### Protein Binding

The extent of nonspecific binding of **1e** and **2a** in murine plasma was determined using rapid equilibrium dialysis. Briefly, commercial mouse K_2_EDTA plasma and PBS were warmed to 37°C, 5% CO_2_, 75% RH in a CO_2_ incubator. The pH was measured and adjusted to pH 7.4 as necessary. Plasma was diluted 20x with PBS and spiked with compounds in DMSO at a final concentration of 5 μM. Two hundred microliters of sample was added to the red chamber of a Pierce Rapid Equilibrium Dialysis (RED) device (ThermoFisher Scientific, Waltham, MA) and 400 μL of PBS to the white chamber in triplicate. Chambers were placed in a plate which was sealed with a semipermeable membrane and incubated for six hours at at 37°C, 5% CO_2_, 75% RH on an orbital shaker. At incubation end, samples (50 μL) were removed from each chamber (PBS side first) and matrix matched prior to addition of an equal volume of methanol containing 0.2% formic acid and 50 ng/ml N-benzylbenzamide) to precipitate protein. Samples were centrifuged twice and evaluated as described above by LC-MS/MS. The extent of protein binding in each matrix was determined as:

$$\%PPB=\frac{C_{R}\;-\;C_{W}}{C_{R}}\times 100\%$$

Where $C_{R}$ is total drug concentration in plasma in red chamber,

$C_{W}$

All concentrations are approximated by peak area ratio of analytes/IS in the LCMS. To account for the impact of dilution, the following correction was applied^40^

$$\mathrm{Undiluted}\;f_{u}=\frac{\backslash \text{raisebox1}ex\$1\$/\backslash \text{raisebox}-1ex\$\backslash \text{varvec}D\$}{\left(\left(\backslash \text{raisebox}1ex\$1\$/\backslash \text{raisebox}-1ex\$\backslash \text{varvec}f\backslash \text{varvec}u_{2}\$\right)\;-\;1\right)\;+\;\backslash \text{raisebox}1ex\$1\$/\backslash \text{raisebox}-1ex\$\backslash \text{varvec}D\$}$$

Where D is the fold dilution and $\backslash \text{varvec}f\backslash \text{varvec}u_{2}$ is the unbound fraction determined using diluted matrix.

### X-ray crystallography and structural analysis

The corresponding region of Yck2 residues 37–345 were PCR amplified from *C. albicans* genomic DNA and subcloned into the vector pMCSG53, which codes for a N-terminal His_6_ tag, TEV protease site, followed by the Yck2 protein. *E. coli BL21* LOBSTR competent cells were transformed with this plasmid and the Yck2 protein was purified using methodology previously described.^41^ Crystallization was performed at RT using the sitting drop method. Crystals of *apo* Yck2 were grown with 1 μL protein at 20 mg/mL and reservoir solution 0.1 M Tris pH 8, 25 mM magnesium chloride and 20% (w/v) PEG3350. To obtain the Yck2–1e, 1f, 2a, and 2b complexes, 1 μL of the inhibitors dissolved in DMSO were soaked into apo Yck2 crystals for 60 minutes. All crystals were cryoprotected in Paratone oil. X-ray diffraction data at 100 K was collected at beamline CMCF-ID, Canadian Macromolecular Crystallography Facility, Canadian Light Source. Diffraction data was reduced using xia2.^42^ The structures were solved by the Molecular Replacement (MR) method using Phenix.phaser^43^ and the Yck2-GW structure (PDB: 6u6a).^12^ Refinement was completed with Phenix.refine and Coot.^44^ All *B*-factors were refined, and TLS parameterization was included in final rounds of refinement. X-ray crystallographic statistics are provided in Table S1. Structural analyses and visualization were completed using PyMOL (Schrödinger).

To generate the Yck2-GW interaction map, The protein structure (PDB: 6U6A) was prepared using the Protein Preparation Wizard in Schrödinger Maestro. Standard preparation protocols were employed, removing waters beyond 5 Å from the co-crystallized ligand, and preparing the structure at a physiological pH (7.4), as well as constraining the convergence of heavy atoms to within 0.3 Å relative to the crystal structure. Notable conformational shifts within the active site following hydrogen-bond network optimization and preparation convergence, include the LYS73 residue which shifted to participate in a hydrogen bonding interaction with HOH514.

### Mouse therapeutic efficacy studies

Female CD-1 mice were rendered neutropenic (neutrophils, < 100/mm^3^) by injecting them with cyclophosphamide (Mead Johnson Pharmaceuticals, Evansville, IN) subcutaneously 4 days (150 mg/kg) and 1 day (100 mg/kg) before infection and 2 days after infection (100 mg/kg). Organisms were subcultured on SDA 24 h prior to infection. The inoculum was prepared by placing three to five colonies into 5 mL of sterile pyrogen-free 0.9% saline that had been warmed to 35°C. The final inoculum was adjusted to a 0.6 transmittance at 530 nm. Disseminated infection was achieved by injection of 1×10^6^ CFU *C*. *albicans* CaLC990 suspended in 0.1 mL inoculum via the lateral tail vein 2 h prior to the start of drug therapy. Six infected animals per treatment group (*n* = 6) were utilized alongside a treatment period of 48–96 h with each molecule at the dose specified in text. At the end of the study period, the animals were sacrificed by CO2 asphyxiation. After sacrifice, the kidneys of each mouse were removed and placed in sterile 0.9% saline at 4°C. The homogenate was then serially diluted 1:10, and aliquots were plated on SDA for viable fungal colony counts after incubation for 24 h at 35°C. The lower limit of detection was 100 CFU/ml. Results were expressed as the mean number of CFU per kidney for three mice. No-treatment and zero-hour controls were included in all experiments. All animal procedures were approved by the Institutional Animal Care and Use Committee at the University of Wisconsin-Madison according to the guidelines of the Animal Welfare Act, The Institute of Laboratory Animals Resources Guide for the Care and Use of Laboratory Animals, and Public Health Service Policy. The approved animal protocol number is DA0042. All mice were maintained at 22.2 °C, 45% humidity, and with light/dark alternating every 12 h.
